# Combined *In Silico* and *In Vitro* Evidence Supporting an Aurora A Kinase Inhibitory Role of the Anti-Viral Drug Rilpivirine and an Anti-Proliferative Influence on Cancer Cells

**DOI:** 10.3390/ph15101186

**Published:** 2022-09-25

**Authors:** Saiful Islam, Theodosia Teo, Malika Kumarasiri, Martin Slater, Jennifer H. Martin, Shudong Wang, Richard Head

**Affiliations:** 1 Drug Discovery and Development, Clinical and Health Sciences, University of South Australia, Adelaide, SA 5000, Australia; 2 Cresset Discovery, New Cambridge House, Litlington, Royston SG8 0SS, UK; 3 Centre for Human Drug Repurposing and Medicines Research, University of Newcastle, Newcastle, NSW 2305, Australia

**Keywords:** repurposing, kinase, cancer, rilpivirine, virtual screening

## Abstract

The global burden of cancer necessitates rapid and ongoing development of effective cancer therapies. One promising approach in this context is the repurposing of existing non-cancer drugs for cancer indications. A key to this approach is selecting the cellular targets against which to identify novel repurposed drugs for pre-clinical analysis. Protein kinases are highly sought-after anticancer drug targets since dysregulation of kinases is the hallmark of cancer. To identify potential kinase-targeted drug candidates from the existing portfolio of non-cancer therapeutics, we used combined *in silico* and *in vitro* approaches, including ligand-based 3D screening followed by biochemical and cellular assessments. This strategy revealed that the anti-viral drug rilpivirine is an Aurora A kinase inhibitor. In view of previous findings implicating Aurora A kinase in abnormal cell cycle regulation, we also examined the influence of rilpivirine on the growth of T47D breast cancer cells. Herein, we detail the identification of rilpivirine as an Aurora A kinase inhibitor, its molecular basis of inhibitory activity towards this kinase, and its Aurora A-mediated anticancer mechanisms in T47D cells. Our results illustrate the value of integrated *in silico* and *in vitro* screening strategies in identifying repurposed drug candidates and provide a scientific basis for further exploring the potential anticancer properties of the anti-viral drug rilpivirine.

## 1. Introduction

Cancer is one of the major worldwide health challenges because of the scale of the disease across populations and its complex pathophysiology. While there exist important therapeutics for treating various types of cancers, there is an ongoing need for additional drugs to improve survival outcomes, minimise adverse side effects, and overcome drug resistance. Although the development of new therapeutics for cancer treatment is to be encouraged, these approaches do have long discovery and evaluation delays, higher safety concerns, and ever-present attrition rates. Further, the high cost of novel anticancer drugs imposes significant economic pressures on the individual patient as well as on the health care systems. However, one additional approach that may potentially expand the armamentarium of drugs for treating cancer involves drug repurposing, which refers to the process of finding new uses for existing approved or clinically advanced drugs albeit in different doses, formulations, or combinations [1]. The general concept of drug repurposing and its potential applications are now well-described elsewhere [2,3,4,5]. However, it is important to recall that drug repurposing stemmed from the poly-pharmaceutical or pleiotropic potential of existing approved drugs. Increasingly, we are appreciating that currently accepted and registered drugs may have more than one biological target and thus show multiple biological activities at different doses [6]. Additionally, different diseases often share common molecular or biological pathways [7]. Increasingly, repurposing is gaining focus as an approach to drug development driven partly by the intrinsic advantages of known pharmacokinetics, well-defined toxicology, and pharmaco-surveillance associated with drugs employed widely in the community [1]. It is also driven by the increasing awareness that diseases are often not homogenous in cell type nor driven by a single gene mutation.

Historically, repurposed drugs were discovered with an element of serendipity or astute clinical observation [4,5]. The advent of advanced *in silico* technologies together with molecular precision associated with cellular biological evaluation has now made it possible to utilise drug- or target-centric approaches to evaluate candidate repurposed drugs in cancer. Of importance, these developments have emerged at the same time as advanced cellular biology has identified key molecular cancer signalling pathways, well-illustrated, for example, by protein kinases [8].

Throughout the last three decades, kinase-targeted drug discovery has become one of the largest areas of focus for the development of cancer therapeutics due in large part to the critical role of kinases in virtually all aspects of cancer biology [9,10]. While there are more than 518 kinases encoded in the human genome, they have highly conserved binding domains in their catalytic regions [8]. This combination of scale in kinase number and conserved active sites favour promiscuity with putative kinase inhibitors. This barrier to specificity offers in theory a potential advantage when examining existing therapeutics as repurposed cancer therapeutics based upon kinase inhibitory properties. Nevertheless, the current repository of kinase-targeted repurposing candidates for cancer treatment is small. Only a few kinase inhibitors have been identified from existing therapeutics to date for potential repurposing, and in most cases, they were identified without any systematic repurposing approaches [1]. This setting provided the stimulus for the current study.

Here, we have utilised a combined approach for identifying putative kinase inhibitors from existing non-cancer therapeutics. First, we employed an *in silico* methodology using ligand-based virtual screening to permit the shortlisting of drug candidates from two commercial databases. During this process, we used a synthetic molecule displaying inhibition of multiple important kinases, namely cyclin-dependent kinases (CDKs), and particularly, CDK9 and Aurora A kinase as the *in silico* ligand. Then, we used cellular and biochemical assays to probe the candidates from the virtual screening analysis. During the course of these experiments, it became evident that the anti-viral drug rilpivirine displayed anticancer activities potentially due to its Aurora A kinase inhibitory properties. Subsequently, we explored the molecular basis of the interaction between rilpivirine and Aurora A kinase and also validated its Aurora A targeted anticancer mechanism using T47D breast cancer cells.

## 2. Results

### 2.1. Identification of Repurposing Drug Candidates Utilising Virtual Screening

**Figure 1:**
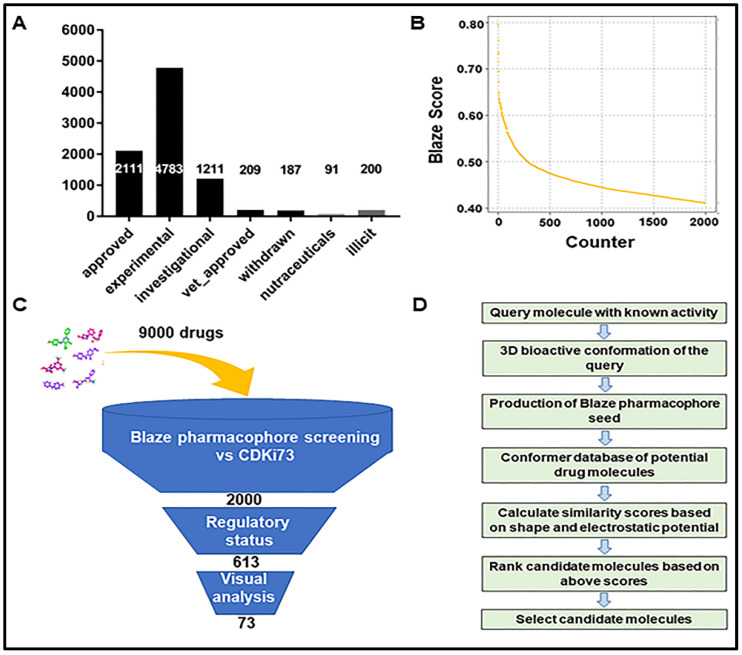
**Virtual screen for identifying potential kinase inhibitors from existing drugs.** (**A**) A summary characterising the properties of the drugs in the commercial databases used for virtual screening. The databases contain 9000 drugs of different regulatory status. (**B**) Illustrating a total of 2000 drug examples selected after the Blaze search. (**C**) A summary cascade employed for identifying virtual screening drug candidates. (**D**) A summary of the workflow using Blaze software.

In our pursuit to identify kinase-targeted repurposing drug candidates, we first employed ligand-based virtual screening of two commercial databases, namely Drug bank and Drug navigator, consisting of about 9000 drugs from different regulatory status (Figure 1A). For this initial screening, we applied an electrostatics and shape-based similarity matching algorithm together with pharmacophore constraints called Blaze (Cresset Discovery Services, Royston, UK). The top-ranking 2000 drug candidates were selected according to the Blaze score (Figure 1B). These 2000 candidates have different regulatory status and diverse target profiles. Based on the regulatory status, 613 candidates already approved or in advanced clinical development stages were selected. Subsequently, this selection was further narrowed using the following criteria: (1) appropriate fit to the binding site of the target of interest, (2) similarity with the known active ligand CDKi73, and (3) no prior annotations associated with kinase activity. These resulted in 73 virtual-screening-based candidates (Supplementary information Table S1). The summary cascade for our virtual screening analysis, as well as the general workflow of the Blaze software used in this screening procedure, can be seen in Figure 1C,D respectively. Then, considering any previously reported anticancer activities and their commercial availability, a set of 24 drug candidates (Supplementary information, Table S2) was selected and purchased for biological testing.

### 2.2. Assessment of Anti-Proliferative Activities of Drug Candidates 

**Figure 2:**
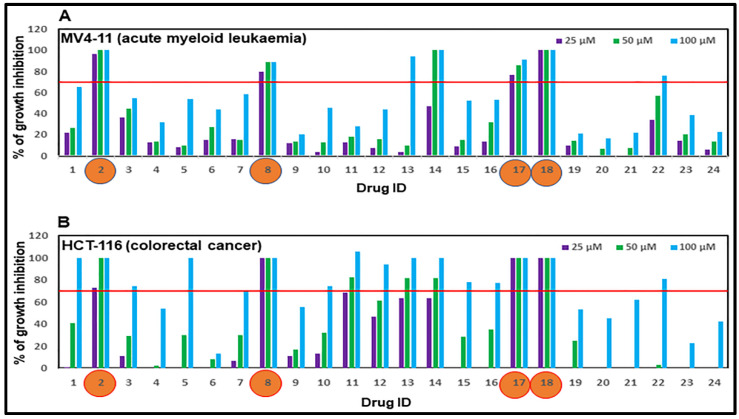
**Anti-proliferative activities of selected drug candidates against (A) acute myeloid leukaemia (MV4-11) and (B) colorectal cancer (HCT-116) cell line.** Cells were treated with each of the 24 candidates for 72 h and the growth inhibitory activities were determined using MTT or resazurin assay for HCT-116 and MV4-11 cell lines, respectively. Drug candidates displayed more than 70% anti-proliferation activity against both cell lines tested at 25 µM concentration are shown in orange circles.

The anti-proliferative properties of the 24 selected drug candidates were assessed us-ing MTT and resazurin assays. We employed a two-stage screening strategy whereby drug candidates were first screened in triplicate at three concentrations, 100, 50, and 25 µM against one haematological cancer cell line (MV4-11 acute myeloid leukaemia) and one solid tumour cell line (HCT116 colorectal carcinoma). We screened the test compounds at high concentrations initially on the basis that, if a response does not occur at these high concentrations, it will not be pursued further at lower concentrations. Notably, four drug candidates showed more than 70% anti-proliferation activity against both the haematological and solid tumour cell lines tested at 25 µM concentration (Figure 2A,B). These included the non-nucleoside reverse transcriptase inhibitors rilpivirine and etravirine, the phosphodiesterase 3 inhibitor pimobendan, and the proton pump inhibitor revaprazan. These four drugs were further tested against a panel of 14 cancer cell lines, and the half-maximal growth inhibition (GI_50_) values were summarised in Table 1.

Etravirine, pimobendan, and rilpivirine exhibited moderate anti-proliferative activities against all 14 cell lines tested with GI_50_ values ranging from 3.045 to 9.450 µM. Revaprazan displayed a mean growth inhibition value of less than 8 µM for ovarian (A2780) and two leukaemia (MV4-11 and MOLM-13) cell lines but was less active against the other 11 cell lines tested (Table 1).

### 2.3. Drug Candidates Displaying Anti-Proliferative Activities Shared Similar Structural Features with the In Silico Ligand

**Figure 3:**
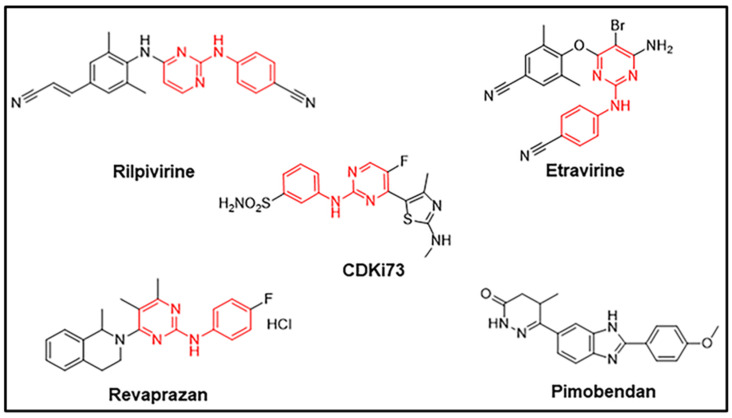
**Structures of the selected candidates based upon their anti-proliferative activities and the structure of CDKi73.** The common core Rilpivirine, Etravirine and Revaprazan share with CDKi73 is highlighted in red.

Upon identification of four drug candidates based on their cellular anti-proliferative potency, their structures were compared with CDKi73, the query molecule used in the *in silico* selection. As seen in Figure 3, despite belonging to different pharmacological classes, namely non-nucleoside reverse transcriptase inhibitors (etravirine and rilpivirine), proton pump inhibitor (revaprazan), and phosphodiesterase 3 inhibitor (pimobendan), all the active drug candidates except pimobendan share a similar *N*-phenylpyrimidin-2-amine core as CDKi73. Structural similarities between the two non-nucleoside reverse transcriptase inhibitors, rilpivirine and etravirine, are also evident (Figure 3).

Based on the observed cellular potency, current clinical use and existing pharmacokinetic data, rilpivirine was selected for follow-up studies. The pharmacological information available for this drug was viewed as helpful. Our focus remained with this drug based upon the observation it displayed Aurora A kinase inhibitory properties as seen immediately below.

### 2.4. Kinase Inhibitory Profiles of Rilpivirine

**Figure 4:**
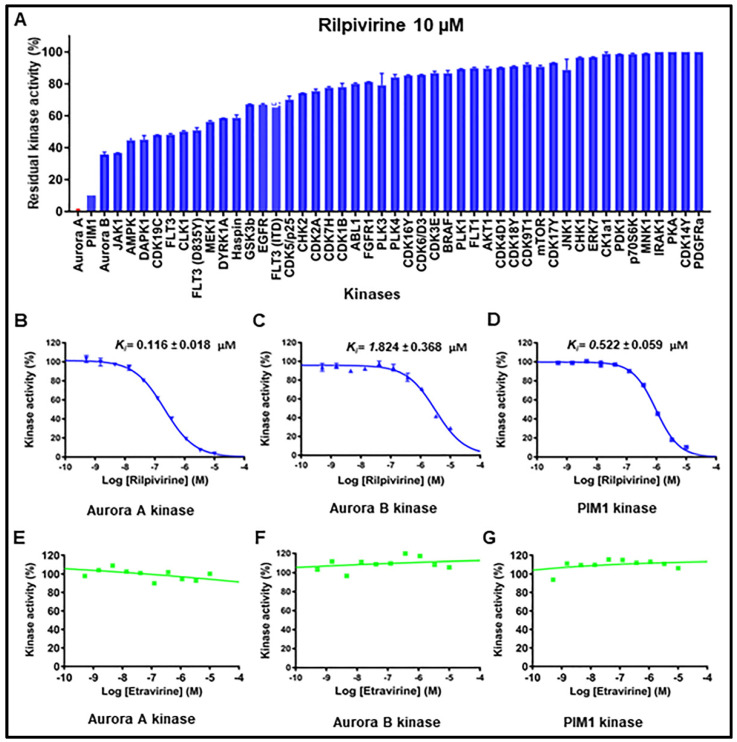
**Kinase inhibition profile of rilpivirine.** (**A**) The effect of rilpivirine on the activity of individual kinases. Rilpivirine, at a concentration of 10 µM, was tested in duplicate. The residual kinase activity refers to the kinase activity remaining after individual kinases were treated with rilpivirine. (**B**–**D**) Dose-response curve of rilpivirine against Aurora A, Aurora B, and PIM1 kinase. (**E**–**G**) Dose–response curve of etravirine against Aurora A, Aurora B and PIM1 kinase. Apparent inhibition constants (*K*_i_) were calculated from IC_50_ values and the appropriate *K*_m_ (ATP) values for each kinase and are represented as mean ± SD derived from four replicates.

To investigate the possible spectrum of kinase inhibitory properties, rilpivirine was tested against a panel of 48 different kinases at 10 µM and the data were expressed as a percentage of residual activity (Figure 4A). The results revealed that rilpivirine was potent against Aurora A kinase with ≤1% residual activity (i.e., ≥99% inhibition) and only one other kinase, PIM1 was inhibited to an extent more than 80%. The activity of Aurora B kinase, a paralogue of Aurora A, was inhibited by 63% when treated with the same concentration of rilpivirine. Subsequently, inhibition constant (*K*_i_) values of rilpivirine for Aurora A, Aurora B and PIM1 kinases were determined to be 0.116, 1.824, and 0.522 µM, respectively (Figure 4B–D).

To probe further the specificity of rilpivirine and to determine whether the activity observed was attributed to its known target effect, etravirine, the other reverse transcriptase inhibitor identified as a candidate in our cellular screening, was also tested against Aurora A, Aurora B, and PIM1 kinases. Remarkably, etravirine does not show any inhibition against these three kinases (Figure 4E–G). These enzymatic data strongly suggest that rilpivirine is a potent inhibitor of Aurora A kinase with appreciable selectivity profile.

### 2.5. Cellular Mode of Action

**Figure 5:**
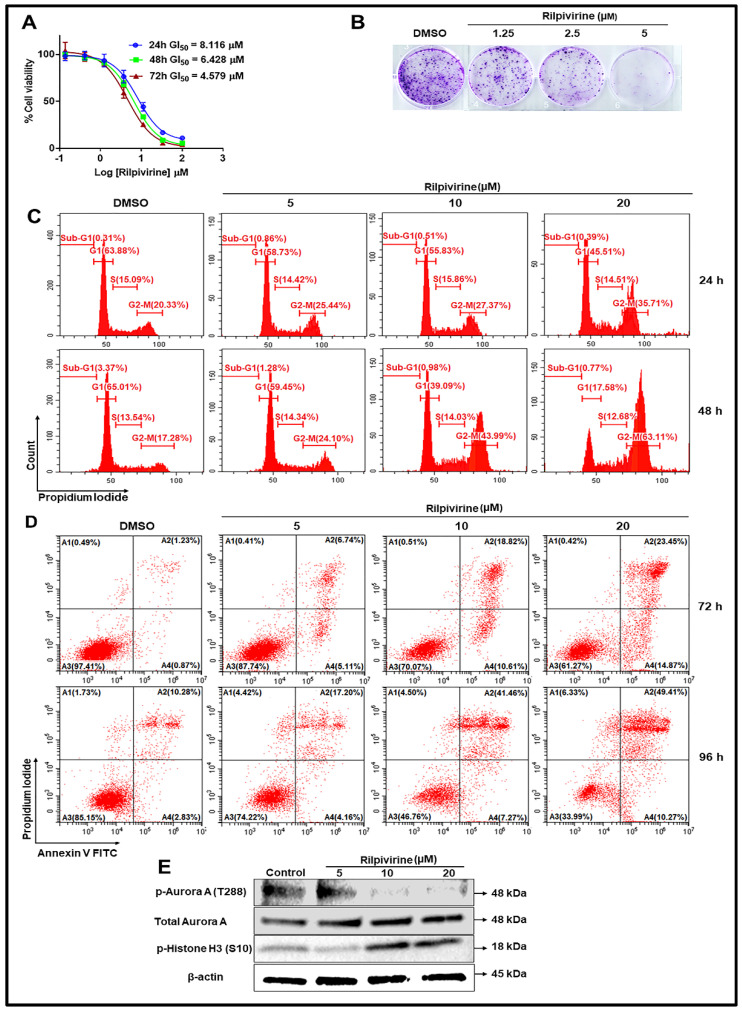
**Cellular mode of action of rilpivirine.** (**A**) Dose–response curve for rilpivirine against T47D cells at three indicated time points. GI_50_ values (µM) are shown. (**B**) Effect of rilpivirine on the formation of colonies in T47D cells. (**C**) Cell cycle analysis of T47D cells after incubation with rilpivirine for 24 or 48 h. Representative histograms with DNA content are presented. (**D**) Induction of apoptosis by rilpivirine in T47D cells after 72 or 96 h treatment. The proportion of apoptotic cells (A2 and A4) was defined as a sum of early apoptotic (annexin V+/PI−) and late apoptotic (annexin V+/PI+) cells. (**E**) Western blot analysis of T47D cells incubated with rilpivirine for 24 h. β-Actin antibody was used as an internal loading control.

Based on the appreciable kinase inhibitory properties, we further evaluated the cellular mode of action of rilpivirine using the Aurora A overexpressing T47D breast cancer cells [11]. For this purpose, T47D cells were treated with different concentrations of rilpivirine for 24, 48, or 72 h and cell viabilities were measured using MTT assays. As shown in Figure 5A, rilpivirine reduced the viability of T47D cells in a time-dependent manner with GI_50_ values of 8.116, 6.428, and 4.579 µM at 24, 48, and 72 h, respectively. We also evaluated the effects of rilpivirine on colony formation of T47D cells. Rilpivirine inhibited the colony-forming ability of T47D cells in a concentration-dependent manner (Figure 5B).

To examine whether the reduction in cell viability was a consequence of cell cycle arrest, the effect of rilpivirine on cell cycle progression was further investigated using flow cytometry. T47D cells were exposed to rilpivirine at 5, 10, or 20 µM for 24 or 48 h and stained with propidium iodide (PI). As shown in Figure 5C, rilpivirine caused a concentration- and time-dependent increase in the population of cells at the G_2_/M phase. Since apoptosis is considered as a distinctive mode of programmed cell death [12], we next quantified the induction of apoptosis by rilpivirine over 72 and 96 h. Compared to the control (vehicle-treated) sample, rilpivirine induced greater apoptosis of T47D cells in a concentration-dependent manner, with the effect being more pronounced at the later time point (Figure 5D). Western blot analysis of T47D cells exposed to rilpivirine at 5, 10, or 20 µM for 24 h also showed an inhibition of autophosphorylation of Aurora A at Thr288 with the total level of Aurora A unaffected and induction of phospho-histone H3 at Ser10 (Figure 5E), confirming the inhibition of cellular Aurora A by rilpivirine.

### 2.6. Binding Hypothesis Determination

**Figure 6:**
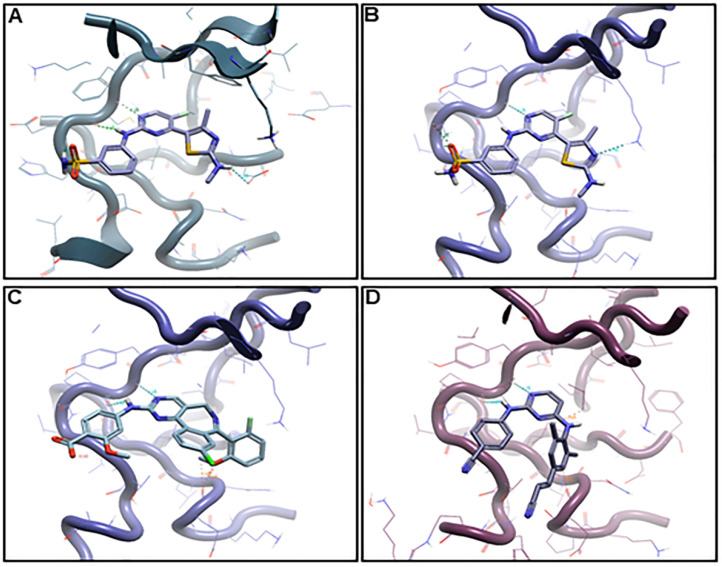
**Proposed binding modes of (A) CDKi73 docked to CDK9 (PDB: 4BCI), (B) CDKi73 docked to Aurora A (PDB: 2X81), (C) alisertib docked to Aurora A (PDB: 2X81), and (D) rilpivirine docked to Aurora A (PDB: 3H0Y)**. Docking was performed manually using the Flare modelling suite (Cresset Discovery Services, Royston, UK). X-ray ligands were chosen based on the closest 2D similarity to the query molecules.

The inhibition of Aurora A kinase was identified as the key anticancer mode of action of rilpivirine. It was, therefore, important to assess the binding characteristics of rilpivirine with this kinase. For this purpose, we conducted conformational analysis using alisertib, the most clinically advanced Aurora A kinase inhibitor as a comparator. Alisertib has been previously reported as displaying the DFG-out conformation during its interaction with Aurora A in its catalytical binding domain [13]. Additionally, we included the query molecule CDKi73 for comparison purposes during the binding hypothesis generation process (manual docking). The proposed binding poses of alisertib, CDKi73, and rilpivirine bound to Aurora A are shown in Figure 6.

As illustrated in Figure 6A,B, CDKi73 aligns within the adenine binding pockets of CDK9 and Aurora A, consistent with its known effects on both kinases. This duality may be attributed to the pyrimidine moiety acting as a ubiquitous kinase hinge binding fragment. In addition, the 2-NH-benzenesulfonamide of CDKi73 is accommodated in both Aurora A and CDK9 albeit by different recognition patterns. The greater influence of CDKi73 on CDK9 compared to that on Aurora A can be assigned to its benzenesulfonamide moiety which is an established key discriminator for potent CDK9 inhibition.

Of major importance are the different binding features of rilpivirine with Aurora A when compared to those of alisertib (Figure 6D,C, respectively), being consistent with their variable activity against Aurora A kinase. Nevertheless, on detailed examination a consistent common feature of the binding of CDKi73, alisertib, and rilpivirine to Aurora A was the interactions involving a pair of hydrogen binding between N1 and 2C-NH of pyrimidine with the backbone amino group of Ala213 at the hinge region of Aurora A (Supplementary Figure S1).

## 3. Discussion

This study identified rilpivirine, the non-nucleoside reverse transcriptase inhibitor, as a potent Aurora A kinase inhibitor. It inhibited the proliferation of a range of cancer cells. Rilpivirine, 4-[[4-[[4-[(1*E*)-2-cyanoethenyl]-2,6-dimethylphenyl]amino]-2-pyrimidinyl]amino]benzonitrile, has been approved for the treatment of human immunodeficiency virus 1 (HIV-1)-infected patients [14]. Compared to other anti-retroviral drugs, it has a more favourable safety profile and similar efficacy at the registered dose. Of importance to this finding is the fact that Aurora A is a member of the serine/threonine kinase. Aurora A and Aurora B are overexpressed or amplified in multiple forms of cancer [15]. Aurora A can act as an oncogene by promoting cellular genomic instability and enhancing cellular proliferation, survival, migration, and invasion [15]. Thus, Aurora A has been identified as a therapeutic target for cancer treatment. Notably, there is no Aurora A kinase inhibitor currently approved as cancer therapeutics. However, a variety of molecules have been shown to inhibit Aurora A kinase and are now in pre-clinical or clinical trials [16]. It is noteworthy that alisertib, used in this study, is the only Aurora A kinase inhibitor that has proceeded to phase III clinical evaluation [16].

It is well-established that Aurora A kinase inhibitors exert their anticancer activity by inhibiting autophosphorylation in the activation loop at Thr288 [17]. This kinase plays a fundamental role in mitotic phases of the cell cycle, especially in regulating the G_2_/M transition of the cell cycle. In our study, rilpivirine displayed the phenotypes consistent with Aurora A inhibition in the T47D breast cancer cell line, which included the accumulation of the G_2_/M cells, inhibition of Aurora A autophosphorylation (pT288) and induction of phospho-histone H3 (S10) [18,19,20]. Rilpivirine, although originally developed as an anti-viral drug, therefore, emerges as a pre-clinical compound of interest in oncology.

An additional key purpose of the current study was to utilise an integrative drug repurposing approach by combining both virtual and cellular biochemical screening. This approach identifies novel kinase inhibitors for anticancer uses from the existing portfolio of non-cancer drugs. Kinases have long been considered an important target for cancer therapeutic development. Accumulating evidence also suggests the utility of kinases as a repurposing drug target [21]. In essence, we have utilised the well-known promiscuity of kinase catalytic sites, which is an appealing region for small molecule targeting, together with the function of kinases involved in cell regulatory processes, many of which are implicated as different hallmarks of cancer and as such provide key anticancer drug targets. Through a series of experiments, we showed that the non-nucleoside reverse transcriptase inhibitor, rilpivirine, is also an Aurora A kinase inhibitor and as such should be explored as a promising repurposing candidate for use in cancer studies. This study and others provided credible evidence that kinase inhibitors can be identified from repurposing existing non-cancer therapeutics. Similar approaches have been reported for the identification of the mammalian target of rapamycin (mTOR) and cyclin-dependent kinase 2 (CDK2) inhibitors [22,23].

Initially, we performed a ligand-centric virtual screen using CDKi73, known to inhibit multiple kinases, especially CDK9 and Aurora A, as a ligand [24]. The hypothesis was that a multi-kinase targeted molecule as a ligand would enhance the chances of identification of an existing therapeutic agent with yet undescribed kinase inhibitory properties. From the 73 virtual screening candidates, 24 hits were purchased and subjected to cellular screening against haematological and solid cancer cell lines, resulting in four active candidates being identified. The cancer cell lines selected were chosen to give a comprehensive representation of the most prevalent types of haematological and solid cancers. After cellular screening, we selected one candidate, i.e., rilpivirine for kinase profiling. Rilpivirine showed the highest potency against Aurora A with a *K*_i_ value of 0.116 µM, and the following two kinases, PIM1 and Aurora B, were about 4.5-fold and 16-fold less potent compared to Aurora A with *K*_i_ values of 0.522 and 1.824 µM, respectively. We further explored whether the anticancer effect of rilpivirine in T47D cell line was mediated by PIM1 kinase inhibition and concluded that the inhibition of PIM1 by rilpivirine is unlikely to contribute to its anticancer property in T47D cell line. This conclusion is based on three observations. First, our 72-h cell viability assay showed that the selective PIM kinase inhibitor AZD1208 inhibits T47D cells at a GI_50_ value of approximately 15 µM (Supplementary Figure S3A). This contrasts with a *K*_i_ for AZD1208 against PIM1 kinase (*K*_i_ < 1 nM) in the cell-free assay [25] and implies that T47D might express a low level of PIM1 protein in this cell line. Consistent with this view is the strong association of HER2 expression with PIM1 and the T47D cells do not express HER2 [26]. Second, we showed that AZD1208 induced G1 arrest of T47D cells (Figure S3B), consistent with that reported previously [25]. In contrast, rilpivirine induced G_2_/M arrest, as did a selective Aurora A kinase inhibitor. Third, PIM1 kinase has been reported to reduce the expression of phospho-histone H3 (S10) [27]. In contrast, we observed that rilpivirine at a concentration up to 20 µM induced phospho-histone H3 (S10), which is also a characteristic phenotype of selective Aurora A kinase inhibitor [20].

Notably, the three other candidates from the cellular screening, i.e., etravirine, pimobendan, and revaprazan, were excluded initially from our follow-up for various reasons. Etravirine has already been reported for anticancer activity due to its role in cyclin D1 downregulation [28]. Pimobendan is a veterinary product and revaprazan, at the time of this experimentation, had not secured FDA approval. It is noteworthy that etravirine, also a non-nucleoside reverse transcriptase inhibitor that shares *N*-phenylpyrimidin-2-amine core with rilpivirine, lacked Aurora A kinase inhibition in our kinase assays. Consistently, etravirine failed to arrest the T47D cells at the G_2_/M phase of the cell cycle (Supplementary Figure S2). These observations suggest a unique structural feature of rilpivirine as an Aurora A kinase inhibitor. However, it is not surprising that a non-nucleoside reverse transcriptase inhibitor may inhibit cancer cell proliferation. Indeed, several inhibitors from this class, including efavirenz and nevirapine, have been extensively studied for their anticancer effects [29,30]. However, their mechanism of anticancer action remains controversial and the involvement of targets other than Aurora kinases, namely inhibition of endogenous reverse transcriptase [31], interaction with the cannabinoid system [32] and oxidative stress in mitochondria [33], have been reported with use of efavirenz and nevirapine. Interestingly, in our cellular screening, rilpivirine showed almost similar anti-proliferative effects in different cell lines studied. Although overexpression of Aurora A has been reported in most cancer cell types, including breast, ovarian, colon, pancreatic and leukaemia [34], we investigated the expression levels of Aurora A in four solid cancer and four leukaemia cell lines and included those cell lines that showed the highest and the lowest sensitivity to rilpivirine. Apart from the HCT-116 cell line that had the lowest sensitivity to rilpivirine, all other cell lines examined showed almost similar Aurora A expression levels (Supplementary Figure S4). There was a trend in two cell lines (MCF-7 and K-562) for a slightly low expression of Aurora A compared to A2780, T47D, NB4, U-937, and HL-60 cell lines. These data explain why the anti-proliferative effects of rilpivirine were similar in different cell lines tested.

During the preparation of this manuscript, an article detailing the *in silico* ligand-centric screening of 2556 approved molecules to find Aurora inhibitors was published [35]. The authors listed several drugs, including a set of anti-viral drugs, i.e., vidarabine, didanosine idoxuridine, ribavirin, and rilpivirine, as potential Aurora A kinase inhibitors. Accordingly, it was appropriate to test the Aurora kinase inhibitory properties of all these drugs. Interestingly, none of these anti-viral drugs except rilpivirine showed any activity against Aurora A (data not shown). The findings of Chakraborty et al. provide *in silico* support for our demonstration of the Aurora A kinase inhibitory properties of rilpivirine. It is important to note that the presence of the *N*-phenylpyrimidin-2-amine moiety in the drugs that inhibited the proliferation of cancer cells does not translate to Aurora A kinase inhibition as does rilpivirine. Detailed analysis based upon docking experimentation further supported this structural specificity.

We explored the molecular basis of the critical interaction of rilpivirine with Aurora A and compared this to alisertib and CDKi73. The binding of rilpivirine in the ATP active site of Aurora A illustrates marked differences when compared to alisertib and CDKi73, rationalising the greater Aurora A kinase inhibitory properties of alisertib (*K*_i_ ≤ 2 nM) and CDKi73 (*K*_i_ ≤ 30 nM) compared with those of rilpivirine (*K*_i_ = 116 nM). There is another aspect of the *in silico* modelling that deserves comment. Using CDKi73 as a comparator, rilpivirine was identified as an inhibitor targeting Aurora A but not CDK9. This is likely due to the presence of the thiazole system at the 4C-position of pyrimidine of CDKi73, a key determinator for its potent CDK9 inhibition. The incorporation of thiazoles within CDK9 inhibitors has been well established by Wang et al. [36], and the absence of this moiety in rilpivirine may account for the selectivity.

In summary, our integrated approaches involving the *in silico* screening with cellular and biochemical assessments led to the identification of rilpivirine as an Aurora A kinase inhibitor. The Aurora A targeted anticancer mode of action of rilpivirine has been confirmed in T47D breast cancer cells. The molecular basis of its Aurora A kinase inhibition has also been determined. Additional preclinical studies are needed, particularly in relation to the prevailing concentration levels of rilpivirine reported for its current indication, which by our preliminary estimates suggest values lower than the mean values seen for inhibition of cancer cell proliferation. There is also a need to understand the *in vivo* pharmacokinetic and pharmacodynamic relationship of rilpivirine before considering any clinical experimentation.

## 4. Materials and Methods

### 4.1. Chemicals

CDKi-73 was synthesised as described previously [37] and kindly provided by Changzhou LeSun Pharmaceuticals Ltd., Changzhou, China. All other drugs were purchased from Targetmol (Boston, MA, USA) or MedChem Express (Monmouth Junction, NJ, USA) with a stated purity of ≥95% by NMR and LC-MS. All drugs were dissolved in dimethyl sulphoxide (DMSO) to a stock solution of 10 mM and then stored at −20 °C.

### 4.2. Cell Culture 

All the cancer cell lines were obtained from the cell bank at the Centre for Drug Discovery and Development, University of South Australia. All cell lines were cultured either in RPMI-1640 (Roswell Park Memorial Institute) or DMEM (Dulbecco’s Modified Eagle’s Medium) supplemented with 10% fetal bovine serum (Thermo Fisher Scientific, Scoresby, VIC, Australia) within a humidified incubator at 37 °C in the presence of 5% CO_2_ following ATCC recommendation. Cell lines were confirmed to be negative for mycoplasma using the MycoAlert^TM^ mycoplasma detection kit (Lonza, Derrimut, VIC, Australia).

### 4.3. Virtual Screening 

The Cresset field-based virtual screening platform, Blaze (formerly known as FieldScreen) [38] was utilised to search the Drug bank and Drug navigator databases to identify compounds similar to the synthetic kinase inhibitor CDKi73. The Blaze search progressed as a multi-tiered approach, including course comparison of field prints and initial clique-based 3D ligand alignment followed by simplex optimisation. The similarity was determined using 50% electrostatic potential [39] and 50% shape [40]. The generated field was then used to screen the databases for other compounds having similar properties and potentially related biological activities. Based on the Blaze score, 2000 top examples were selected and further filtered to generate a set of 73 virtual screening-based candidates.

### 4.4. Cell Viability Assays (MTT and Resazurin Assays)

The MTT (3-(4,5-dimethylthiazol-2-yl)-2,5-diphenyl tetrazolium bromide; Sigma-Aldrich, St Louis, MO, USA) assay was used to determine half-maximal growth inhibition values (GI_50_) in response to drug treatment using a number of different adherent cancer cell lines. The MTT was converted by metabolically active cells into an insoluble formazan product which was solubilised with 100% DMSO. The solubilised formazan gives absorbance at 560 nm that is directly proportional to the number of viable cells. The assay was carried out as previously described [41]. The absorbance of treated wells was read using an EnVision multi-label plate reader. The resazurin assay was carried out for non-adherent cell lines as reported previously [42]. GI_50_ values were determined by non-linear regression analysis using GraphPad Prism software version 6.0 (San Diego, CA, USA).

### 4.5. Colony Formation Assay

A total of 1500 cells/well were plated on a 6-well plate and allowed to adhere for 6–8 h. Appropriate concentrations of rilpivirine were added, and then the cells were put back into the incubator for 14 days. The medium was changed every 3 days. After the incubation, the cells were washed with 1 mL PBS carefully. Cells were then fixed with 1% formaldehyde and stained with crystal violet. Images were taken using a camera fitted with a microscope.

### 4.6. Kinase Assays

Kinase inhibitory profiles of drug candidates were measured using ADP-Glo^TM^ assay kits (Promega, Madison, WI, USA) or externally using radioisotope-based assays (Reaction Biology Corporation, Malvern, PA, USA). The ADP-Glo assay was performed as reported previously [43]. The assay plate was read for luminescence using EnVision multi-label plate reader (PerkinElmer, Waltham, MA, USA). Half-maximal inhibition (IC_50_) values were determined from a plot of percent residual activity versus concentration of test compounds using GraphPad Prism software. *K*_i_ values were calculated from IC_50_ values using the Cheng–Prusoff equation: *K*_i_ = IC_50_/[1 + ([ATP]/*K*_m_(ATP))], where [ATP] is the ATP concentration used for the IC_50_ determination and *K*_m_(ATP) for each kinase is determined from an individual experiment [41,44].

### 4.7. Cell Cycle Analysis

The effect of rilpivirine on cell cycle distribution in T47D breast cancer cells was evaluated by flow cytometric analysis. Briefly, cells were seeded in 6-well plates at a density of 6 × 10^4^ cells/well and then plates were incubated overnight at 37 °C in a 5% CO_2_ incubator. Following the incubation, the test compound was added to individual wells and the plates were incubated for 48 and 72 h. Subsequently, the medium was removed from the wells and transferred into fluorescence-activated cell sorting (FACS) tubes. The adherent cells remaining were trypsinised, re-suspended in media, and transferred into the FACS tubes. Cells were centrifuged, fixed with cold 70% ethanol for 15 min, and pelleted. The pelleted cells were then re-suspended in 200 μL propidium iodide (PI) solution (50 μg/mL propidium iodide, 0.1 mg/mL ribonuclease A, 0.1% sodium citrate, 0.1% Triton X-100) and incubated in the dark for 1.5 h at room temperature. Following the incubation, 200 μL PBS was added. Samples were assessed using a flow cytometer (Cytoflex; Beckman Coulter Inc, Brea, CA, USA), and data were analysed using the CytExpert software version 2.1 (Beckman Coulter, Brea, CA, USA).

### 4.8. Apoptosis Analysis

Induction of apoptosis was examined using Annexin-V FITC/PI stains and a similar experimental procedure for the cell cycle was followed apart from the apoptosis analysis, which did not require fixation of cells with 70% ethanol. Instead of ethanol fixation, the cell number of each sample was counted, and the cells were diluted to 1 × 10^5^ cells with 1 mL PBS in fresh FACS tubes. Cells were centrifuged and then resuspended in 1 mL of cold PBS (the whole step was repeated twice). Pelleted cells were resuspended in 100 μL binding buffer, and subsequently, 3 μL of Annexin V and 3 μL of PI were added to all tubes and incubated in the dark for 15 min at room temperature. Following the incubation period, 200 μL of 1 x binding buffer was added. Samples were assessed using a Cytoflex flow cytometer (Beckman Coulter, Brea, CA, USA) within 1 h of staining and data were analysed using CytExpert software version 2.1 (Beckman Coulter, Brea, CA, USA).

### 4.9. Western Blot Analysis

Western blotting was performed as described previously [45]. Briefly, cells (8 × 10^5^) were seeded in a culture dish with 10 mL medium and incubated overnight at 37 °C, 5% CO_2_. Untreated and compound-treated cells were lysed using phosphate lysis buffer and protease inhibitors. The protein concentration of each sample was determined by Bio-Rad DC^TM^ protein assay (Bio-Rad Laboratories, Hercules, CA, USA). The protein was deactivated at 95 °C for 5 min and then resolved on 4–20% polyacrylamide gels by electrophoresis. Proteins were transferred to polyvinylidene difluoride (PVDF) membrane and blocked for 1 h with 10% skimmed milk (SM) in Tris-buffered saline and Tween (TBST). After adding the primary antibody, the membranes were incubated overnight on a rocker in a 4 °C cold room. The following day, membranes were washed in TBST (4 × 20 min) and incubated for at least 1 h at room temperature with the appropriate horseradish peroxidase-conjugated secondary antibody. Following this, the blots were washed with TBST (4 × 20 min) again. The blots were then treated with Western blotting detection reagent and the band intensity was determined using a Bio-Rad ChemiDoc^TM^ MP imaging system (Bio-Rad Laboratories). All the antibodies used for protein detection were from Cell Signaling Technology (Danvers, MA, USA).

### 4.10. Molecular Modelling

Binding hypotheses determination based upon manual docking was utilised. This made use of crystallographic bound ligands and the corresponding protein conformation as a more appropriate molecular reference for the bound geometry for the queries. The X-ray ligands which were closest in chemical 2D similarity to the query molecules were chosen. Molecular superpositions of kinase inhibitors were established by producing a super alignment of a number of reference kinase protein structures, using Cresset’s protein modelling suite ‘Flare’ with PDBs: 2X81, 3H0Y and 4BCI, contrasting and comparing the displays of the crystallographic inhibitors. Relevant ligands were manually built in 3D, minimised, and modelled by adjusting torsions to produce conformations approximating the closest relevant bound reference ligands. Each ligand example was then locally minimised in the context of the appropriate protein, allowing some but only small residue movements as the ligands themselves were minimised.

### 4.11. Data Analysis

Microsoft Excel (Redmond, Washington, DC, USA) or GraphPad Prism version 6.0 (San Diego, CA, USA) was used for data analysis. GI_50_ values were determined through performing curve-fitting and calculated using a four-parameter logistic non-linear regression model.

## 5. Conclusions

The current study provides *in silico* and *in vitro* support for Aurora A kinase inhibitory role of the anti-viral drug rilpivirine. This study also provides some insights into the anticancer effects of rilpivirine. The approach presented in this study can potentially be applied more broadly to identify other new kinase-targeted non-cancer therapeutics to be repurposed for cancer treatment.

## Figures and Tables

**Table 1:** Anti-proliferative activities of etravirine, pimobendan, rilpivirine, and revaprazan against a panel of human solid cancer and leukaemia cell lines using MTT and resazurin assay, respectively. Data shown are the half-maximal growth inhibition (GI_50_) values with standard deviations.

Human Cancer Cell Lines		72 h GI_50_ (µmol/L) ± SD			
Origin	Destination	Etravirine	Pimobendan	Rilpivirine	Revaprazan
**Breast**	**MCF-7**	5.174 ± 0.373	5.939 ± 0.085	5.928 ± 0.206	11.02 ± 0.675
**Colon**	**HCT-116**	5.753 ± 0.520	5.450 ± 0.658	9.422 ± 0.812	17.60 ± 3.020
	**HT-29**	5.210 ± 0.634	6.121 ± 1.828	5.195 ± 0.469	11.42 ± 1.920
**Ovarian**	**A2780**	4.101 ± 1.266	4.514 ± 1.009	3.045 ± 0.823	6.301 ± 1.629
**Pancreatic**	**PANC-1**	5.185 ± 0.256	7.195 ± 0.168	4.764 ± 0.340	14.04 ± 0.851
**Leukaemia**	**MV4-11**	5.863 ± 1.121	3.788 ± 0.699	4.375 ± 0.516	7.926 ± 0.983
	**MOLM-13**	7.052 ± 1.142	4.053 ± 1.811	4.266 ± 0.644	7.828 ± 0.715
	**JURKAT**	7.785 ± 0.408	4.756 ± 0.224	4.281 ± 0.767	32.67 ± 4.428
	**HL-60**	5.952 ± 0.473	5.254 ± 0.202	4.762 ± 1.619	38.06 ± 1.607
	**K-562**	8.163 ± 0.602	7.033 ± 0.165	6.858 ± 0.745	25.37 ± 0.926
	**NB4**	4.782 ± 0.341	4.062 ± 0.494	3.395 ± 1.720	42.35 ± 3.673
	**PL-21**	4.465 ± 0.282	4.535 ± 0.226	3.737 ± 0.636	16.55 ± 1.232
	**U-937**	4.794 ± 0.497	3.782 ± 0.138	3.628 ± 1.939	28.14 ± 2.887
	**THP-1**	9.450 ± 0.730	3.118 ± 0.377	6.032 ± 0.394	18.15 ± 1.884

Values are the mean of at least three replicates.

## Data Availability

Data is contained within the article and supplementary material.

## Supplementary Materials

The following supporting information can be downloaded at: https://www.mdpi.com/article/10.3390/ph15101186/s1, Table S1: List of virtual screening-based drug candidates; Table S2: List of biologically tested drug candidates; Figure S1: Predicted binding modes of CDKi73, alisertib, and rilpivirine to Aurora A. Figure S2: Cell cycle analysis of T47D cells after treatment with etravirine (10 µM) for 48 h; Figure S3: (A) dose–response curve for AZD1208 against T47D cells at 72 h; (B) cell cycle analysis of T47D cells after incubation with AZD1208 (30 µM) or rilpivirine (10 µM) for 48 h; Figure S4: Aurora A protein expression across a panel of solid cancer and leukaemia cell lines with varying sensitivity to rilpivirine.
