# Supplementary material for: Combined In Silico and In Vitro Evidence Supporting an Aurora A Kinase Inhibitory Role of the Anti-Viral Drug Rilpivirine and an Anti-Proliferative Influence on Cancer Cells
Source: Pharmaceuticals (Basel). 2022 Sep 25;15(10):1186. doi: 10.3390/ph15101186 (PMC9607353; doi:10.3390/ph15101186)
Supplement: Supplementary file 1 [file pharmaceuticals-15-01186-s001.zip › pharmaceuticals-1834603 Supplementary.pdf]

Supporting Information

Combined *in silico* and *in vitro* evidence supporting an Aurora-A kinase inhibitory role of the anti-viral drug rilpivirine and an anti-proliferative influence on cancer cells

Saiful Islam[1], Theodosia Teo[1], Malika Kumarasiri[1], Martin Slater[3], Jennifer H. Martin[2], Shudong Wang[1], Richard Head[1*]

[1]Drug Discovery and Development, Clinical and Health Sciences, University of South Australia Adelaide SA 5000, Australia.

[2]Centre for Human Drug Repurposing and Medicines Research, University of Newcastle, NSW 2305, Australia.

[3]Cresset Discovery, New Cambridge House, Litlington, Royston, SG8 0SS, United Kingdom.

*Correspondence: Emeritus Professor Richard Head, Drug Discovery and Development, Clinical and Health Sciences, University of South Australia, Adelaide SA 5000, Australia.

E-mail: richard.head@unisa.edu.au

Table S1: List of virtual screening-based drug candidates

	Structure	Name	Known pharmacological targets	Indication	BLAZE_ Score
1	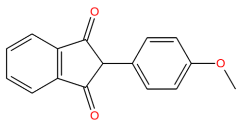	Anisindione	Vitamin K-dependent gamma-carboxylase	Prophylaxis and treatment of venous thrombosis and its extension, the treatment of atrial fibrillation with embolization, the prophylaxis and treatment of pulmonary embolism, and as an adjunct in the treatment of coronary occlusion.	0.5696
2	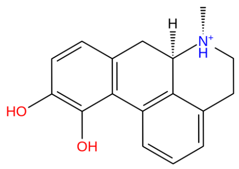	Apomorphine	Dopamine receptors D(2), D(3), D(4)	Acute, intermittent treatment of hypomobility, off episodes (end-of-dose wearing off and unpredictable on/off episodes) associated with advanced Parkinson's disease.	0.4362
3	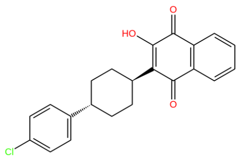	Atovaquone	Bacterial Cytochrome b, Bacterial Dihydroorotate dehydrogenase	*Pneumocystis carinii* pneumonia in patients who are intolerant to trimethoprim-sulfamethoxazole (TMP-SMX). Also indicated for the acute mild to moderate PCP in patients who are intolerant to TMP-SMX.	0.442
4	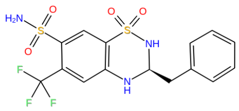	Bendroflumethiazide	Solute carrier family 12 member 3, Calcium-activated potassium channel subunit alpha-1	High blood pressure and management of edema related to heart failure.	0.4873
5	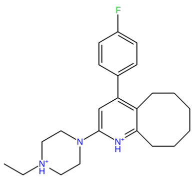	Blonanserin	D2 receptor; 5-HT2 receptor	Schizophrenia	0.4399

	Structure	Name	Known pharmacological targets	Indication	BLAZE_ Score
6	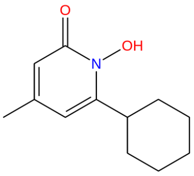	Ciclopirox	Sodium/potassium-transporting ATPase subunit alpha-1	Immunocompetent patients with mild to moderate onychomycosis of fingernails and toenails without lunula involvement, due to *Trichophyton rubrum*	0.4337
7	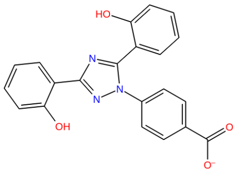	Deferasirox	Iron chelator	Transfusional hemosiderosis	0.4621
8	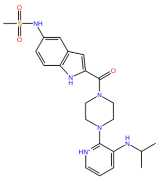	Delavirdine	Reverse transcriptase/RNase H	HIV-1 infection in combination with appropriate antiretroviral agents	0.47
9	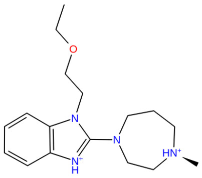	Emedastine	Histamine H1 receptor	Allergic conjunctivitis	0.4223
10	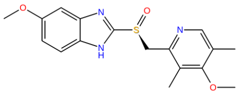	Esomeprazole	Potassium-transporting ATPase alpha chain 1	Acid-reflux disorders (GERD), peptic ulcer disease, H. pylori eradication, and prevention of gastroinetestinal bleeds with NSAID use.	0.5953
11	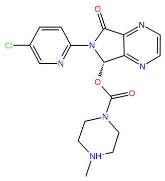	Eszopiclone	Gamma-aminobutyric acid receptor subunit alpha-1, Translocator protein, GABA-A receptor	Insomnia	0.4526

	Structure	Name	Known pharmacological targets	Indication	BLAZE_ Score
12	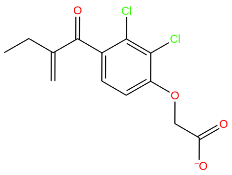	Ethacrynic acid	Solute carrier family 12 member 1, Sodium/potassium-transporting ATPase subunit alpha-1	High blood pressure and edema caused by diseases like congestive heart failure, liver failure, and kidney failure.	0.4398
13	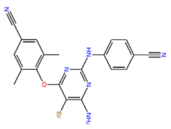	Etravirine	HIV-1 reverse transcriptase	HIV-1 infections resistant to therapy with other NNRTIs and antiretroviral agents.	0.4982
14	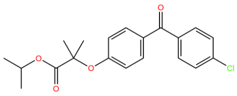	Fenofibrate	Peroxisome proliferator-activated receptor alpha	Reduce elevated LDL-C, Total-C,Triglycerides and Apo B, and to increase HDL-C in adult patients with primary hypercholesterolemia or mixed dyslipidemia (Fredrickson Types IIa and IIb)	0.4275
15	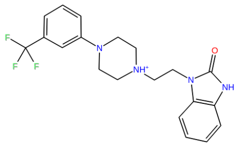	Flibanserin	5-hydroxytryptamine receptor 1A and 2A, D(4) dopamine receptor	Hypoactive sexual desire disorder (HSDD) in premenopausal women.	0.4118
16	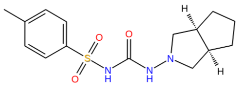	Gliclazide	ATP-binding cassette sub-family C member 8	Reduce hyperglycemia in type 2 diabetes mellitus.	0.4187
17	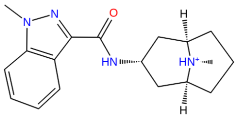	Granisetron	5-hydroxytryptamine receptor 3A	Nausea and vomiting associated with initial and repeat courses of emetogenic cancer therapy (including high dose cisplatin), postoperation, and radiation.	0.4624

	Structure	Name	Known pharmacological targets	Indication	BLAZE_ Score
18	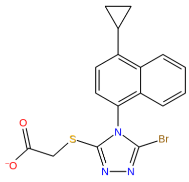	Lesinurad	Solute carrier family 22 members 11 and 12	Hyperuricemia associated with gout in patients who have not achieved target serum uric acid levels with a xanthine oxidase inhibitor alone.	0.4695
19	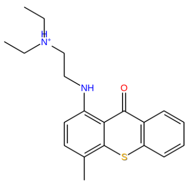	Lucanthone	DNA topoisomerase 2-alpha, DNA-(apurinic or apyrimidinic site) lyase, DNA, DNA topoisomerase 1	Radiation sensitizer in the treatment of brain cancer.	0.4272
20	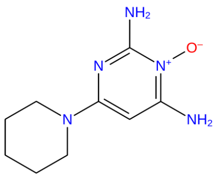	Minoxidil	ATP-sensitive inward rectifier potassium channel 1	Severe hypertension and in the topical treatment (regrowth) of androgenic alopecia in males and females and stabilisation of hair loss in patients with androgenic alopecia.	0.4726
21	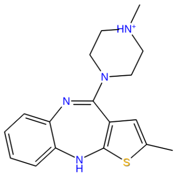	Olanzapine	5-hydroxytryptamine receptor 2A, D(2) dopamine receptor	Acute and maintenance treatment of schizophrenia and related psychotic disorders, as well as acute treatment of manic or mixed episodes of bipolar 1 disorder.	0.4797
22	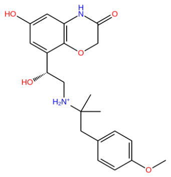	Olodaterol	Beta-2 adrenergic receptor	Chronic obstructive pulmonary disease (COPD), including chronic bronchitis and/or emphysema.	0.4367
23	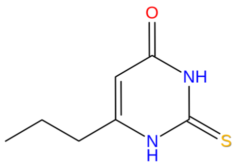	Propylthiouracil	Thyroid peroxidase	Hyperthyroidism	0.4544

	Structure	Name	Known pharmacological targets	Indication	BLAZE_Score
24	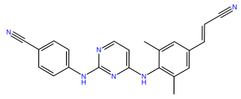	Rilpivirine	Reverse transcriptase/RNase H	HIV-1 infections in combination with at least 2 other antiretroviral agents.	0.5593
25	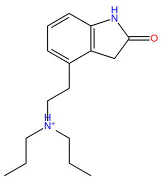	Ropinirole	D(2) and D(3) dopamine receptors	Idiopathic Parkinson's disease. Restless legs syndrome.	0.447
26	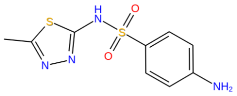	Sulfamethazine	Dihydropteroate synthase	Human and animal urinary tract infection	0.4271
27	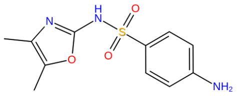	Sulfamoxole	Dihydropteroate synthetase	Bacterial infection.	0.4649
28	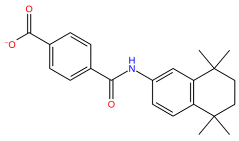	Tamibarotene	Retinoic acid receptor alpha	Relapsed or refractory acute promyelocytic leukemia (APL)	0.4618
29	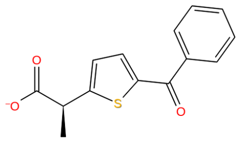	Tiaprofenic acid	Prostaglandin G/H synthase 1 and 2	Pain, arthritic pain.	0.4309
30	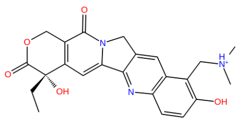	Topotecan	DNA topoisomerase 1, DNA	Ovarian, cervical cancer	0.4627
31	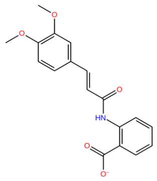	Tranilast	Unknown	Bronchial asthma, keloid and hypertrophic scar, and allergic disorders such as asthma, allergic rhinitis and atopic dermatitis.	0.4536

	Structure	Name	Known pharmacological targets	Indication	BLAZE_ Score
32	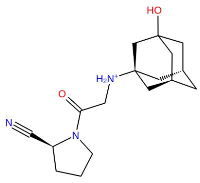	Vildagliptin	Dipeptidyl peptidase 4	Reduce hyperglycemia in type 2 diabetes mellitus.	0.5172
33	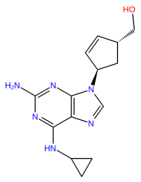	Abacavir	Reverse transcriptase/RNase H	HIV-1 infection, in combination with other antiretroviral agents.	0.4664
34	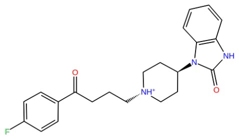	Benperidol	Not available	Phycotic disorders.	0.4293
35	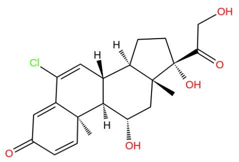	Cloprednol	Not available	Not available	0.470
36	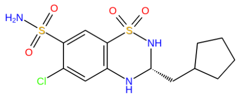	Cyclopenthiazide	Not available	Not available	0.4731
37	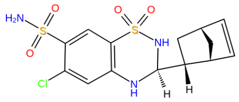	Cyclothiazide	Sodium/potassium-transporting ATPase subunit gamma	Adjunctive therapy in edema associated with congestive heart failure, hepatic cirrhosis, and corticosteroid and estrogen therapy. It is also indicated in the management of hypertension either as the sole therapeutic agent or to enhance the effectiveness of other drugs.	0.4814

	Structure	Name	Known pharmacological targets	Indication	BLAZE_ Score
38	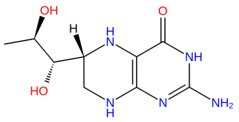	Dapropterin	Endothelial nitric oxide synthase, Phenylalanine-4-hydroxylase, Tyrosine 3-monooxygenase, Tryptophan 5-hydroxylase 1	Tetrahydrobiopterin (BH4) deficiency.	0.4799
39	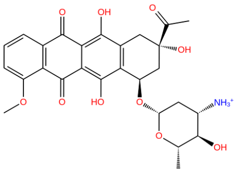	Daunorubicin	DNA, DNA topoisomerase 2-alpha	Acute nonlymphocytic leukemia (myelogenous, monocytic, erythroid) of adults and acute lymphocytic leukemia of children and adults.	0.4425
40	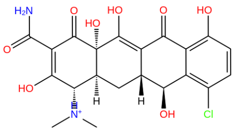	Demeclocycline	30S ribosomal proteins S4 and S9	Lyme disease, acne, and bronchitis. Also indicated (but rarely used) to treat urinary tract infections, gum disease, malaria, and other bacterial infections such as gonorrhea and chlamydia.	0.4419
41	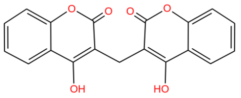	Dicumarol	Vitamin K epoxide reductase complex subunit 1	Decreasing blood clotting. Often used along with heparin for treatment of deep vein thrombosis.	0.4487
42	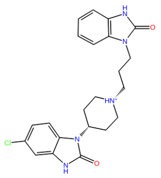	Domperidone	D(2) and D(3) dopamine receptors	Dyspepsia, heartburn, epigastric pain, nausea, and vomiting.	0.4412
43	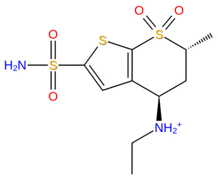	Dorzolamide	Carbonic anhydrase 2	Open-angle glaucoma	0.4367

	Structure	Name	Known pharmacological targets	Indication	BLAZE_ Score
44	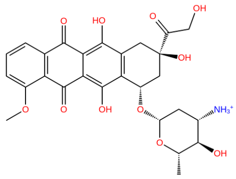	Doxorubicin	DNA, DNA topoisomerase 2-alpha	Disseminated neoplastic conditions	0.4567
45	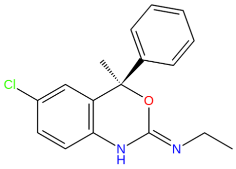	Etifoxine	Not available	Not available	0.4465
46	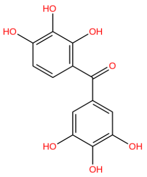	Exifone	Not available	Not available	0.4724
47	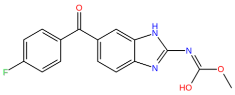	Flubendazole	disruption of microtubule structure and function	Antiprotozoal	0.4918
48	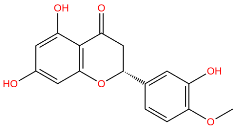	Hesperetin	Microsomal triglyceride transfer protein large subunit, Sterol O-acyltransferase 1	Lowering cholesterol and, possibly, otherwise favorably affecting lipids	0.4942
49	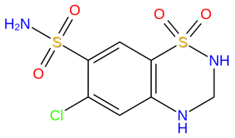	Hydrochlorothiazide	Solute carrier family 12 member 3	High blood pressure and management of edema.	0.4321

	Structure	Name	Known pharmacological targets	Indication	BLAZE_ Score
50	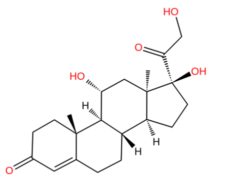	Hydrocortisone	Glucocorticoid receptor, Annexin A1	Inflammatory and pruritic manifestations of corticosteroid-responsive dermatoses. Also used to treat endocrine (hormonal) disorders (adrenal insufficiency, Addisons disease). It is also used to treat many immune and allergic disorders, such as arthritis, lupus, severe psoriasis, severe asthma, ulcerative colitis, and Crohn's disease.	0.4949
51	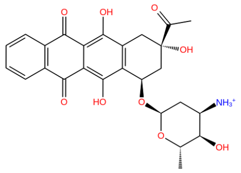	Idarubicin	DNA, DNA topoisomerase 2-alpha	Acute myeloid leukemia (AML) in adults.	0.480
52	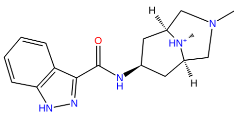	Indisetron	5-HT3 and 5-HT4 receptor	Prophylaxis of chemo therapy-induced nausea and vomiting	0.4868
53	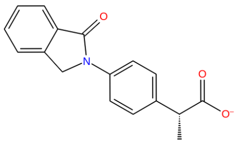	Indoprofen	Not available	Analgesic and anti-inflammatory	0.4844
54	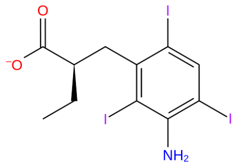	Iopanoic acid	Inhibitors of thyroid hormone	Radiocontrast medium	0.4389

	Structure	Name	Known pharmacological targets	Indication	BLAZE_ Score
55	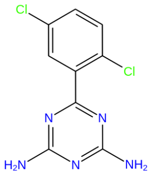	Irsogladine	Phosphodiesterase inhibitor	Peptic ulcer disease and acute gastritis	0.5156
56	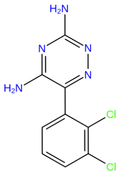	Lamotrigine	Sodium channel protein type 2 subunit alpha	Partial seizures in epilepsy and generalized seizures of Lennox-Gastaut syndrome. Also for the maintenance treatment of bipolar I disorder and depression.	0.412
57	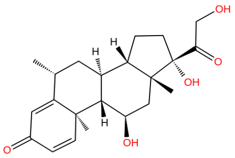	Methylprednisolone	Glucocorticoid receptor	Adjunctive therapy for short-term administration in rheumatoid arthritis.	0.4888
58	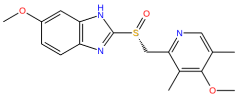	Omeprazole	Potassium-transporting ATPase alpha chain 1	Duodenal ulcers, benign gastric ulcers, gastroesophageal reflux disease (GERD), heartburn and other symptoms associated with GERD, erosive esophagitis, and long-term treatment of pathological hypersecretory conditions like Zollinger-Ellison syndrome, multiple endocrine adenomas, and systemic mastocytosis.	0.5433
59	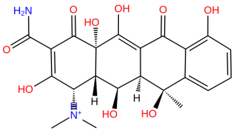	Oxytetracycline	30S ribosomal proteins S4 and s9	Infections caused by a variety of Gram-positive and Gram-negative microorganisms	0.4785

	Structure	Name	Known pharmacological targets	Indication	BLAZE_ Score
60	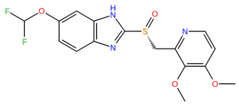	Pantoprazole	Potassium-transporting ATPase alpha chain 1	Short-term (up to 16 weeks) treatment of erosive esophagitis.	0.5832
61	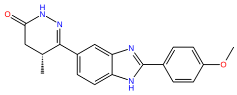	Pimobendan	Phosphodiesterase 3 (PDE3)	Management of heart failure in dogs	0.4189
62	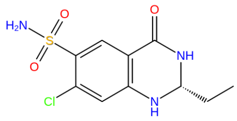	Quinethazone	Carbonic anhydrases 1 and 2. Solute carrier family 12 members 1, 2 and 3.	Hypertension.	0.5282
63	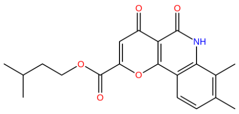	Repirinast	Inhibit histamine release	Antiallergic	0.5012
64	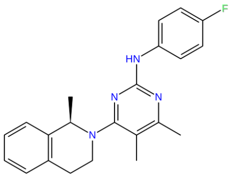	Revaprazan	Proton pump inhibitor	Duodenal ulcer, gastric ulcer and gastritis	0.6037
65	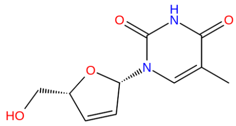	Stavudine	Reverse transcriptase/RNase H	Human immunovirus (HIV) infections.	0.4786
66	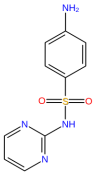	Sulfadiazine	Bacterial dihydropteroate synthetase	Rheumatic fever and meningococcal meningitis	0.4786

	Structure	Name	Known pharmacological targets	Indication	BLAZE_ Score
67	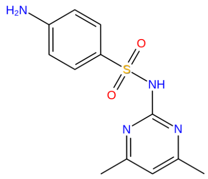	Sulfamethazine	Bacterial dihydropteroate synthetase	Bacterial infections causing bronchitis, prostatitis and urinary tract infections.	0.4909
68	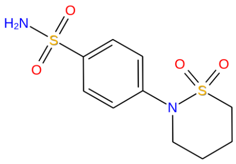	Sulthiame	Inhibition of carbonic anhydrase and blocking of sodium channels	An anticonvulsant for behavioural disorders	0.5179
69	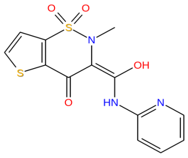	Tenoxicam	Prostaglandin G/H synthase 2	Rheumatoid arthritis, osteoarthritis, backache, and pain.	0.4307
70	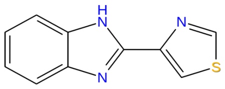	Thiabendazole	Fungal and parasitic fumarate reductase flavoprotein subunit	Strongyloidiasis (threadworm), cutaneous larva migrans (creeping eruption), visceral larva migrans, and trichinosis.	0.5478
71	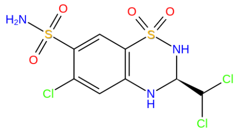	Trichlormethiazide	Solute carrier family 12 member 1, Sodium/potassium-transporting ATPase subunit alpha-1	oedema (including that associated with heart failure) and hypertension.	0.4989
72	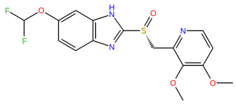	Pantoprazole	Potassium-transporting ATPase alpha chain 1	Short-term (up to 16 weeks) treatment of erosive esophagitis.	0.5812
73	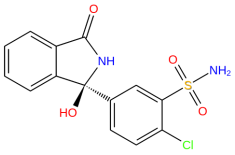	Chlorthalidone	Solute carrier family 12 member 1	hypertension.	0.5073

Table S2: List of biologically tested drug candidates

Drug ID	Name of drug candidate	Drug ID	Name of drug candidate
1	Esomeprazole	13	Dicumarol
2	Etravirine	14	Domperidone
3	Fenofibrate	15	Omeprazole
4	Granisetron	16	Pantoprazole
5	Lesinurad	17	Pimobendan
6	Minoxidil	18	Revaprazan
7	Olanzapine	19	Sulfadiazine
8	Rilpivirine	20	Sulfamethazine
9	Sulfamethizole	21	Tenoxicam
10	Tranilast	22	Trichlormethiazide
11	Vildagliptin	23	Chlorthalidone
12	Abacavir	24	Gliclazide

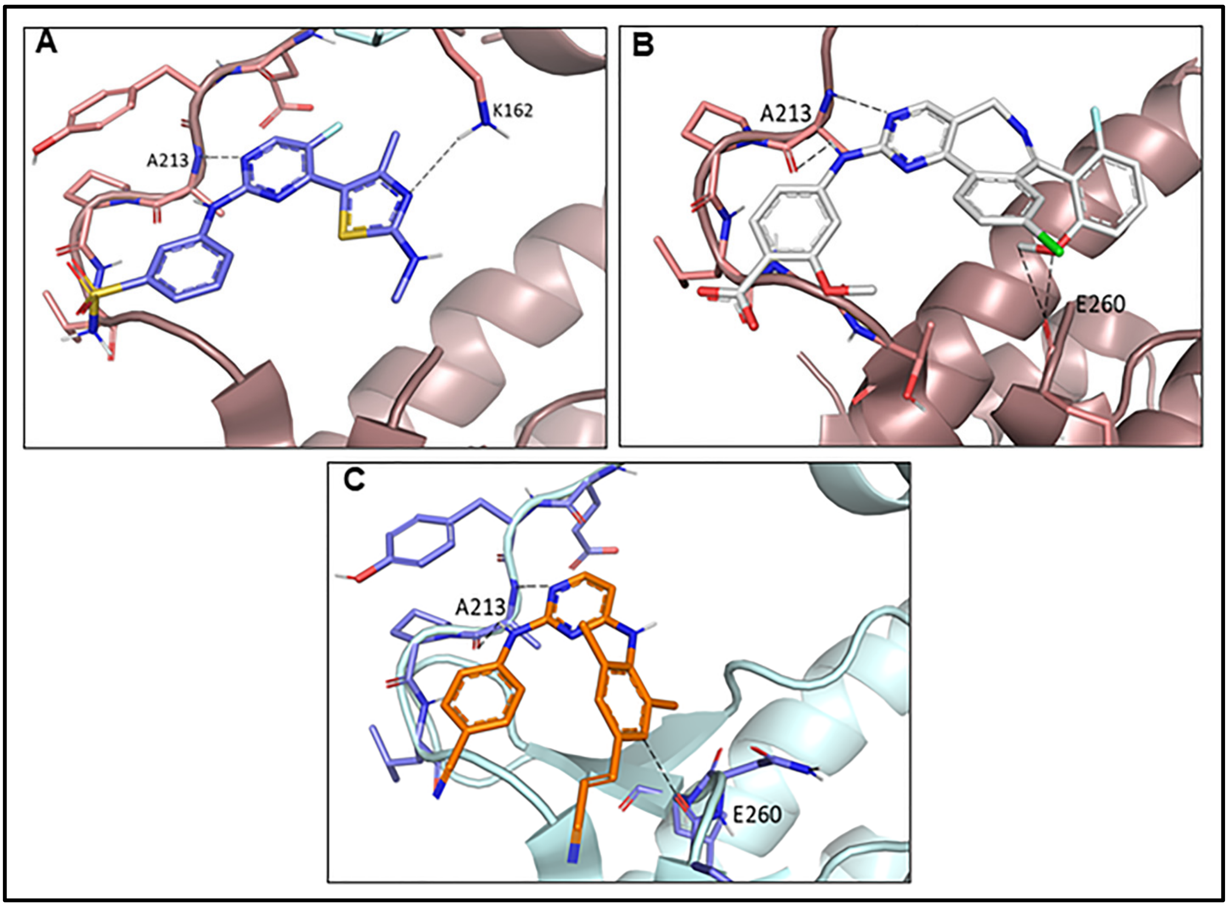

Figure S1: Predicted binding modes of CDKi73 docked to Aurora-A (PDB: 2X81) (A), alisertib docked to Aurora-A (PDB: 2X81) (B) and rilpivirine docked to Aurora-A (PDB: 3H0Y)(C). Critical residues are annotated.

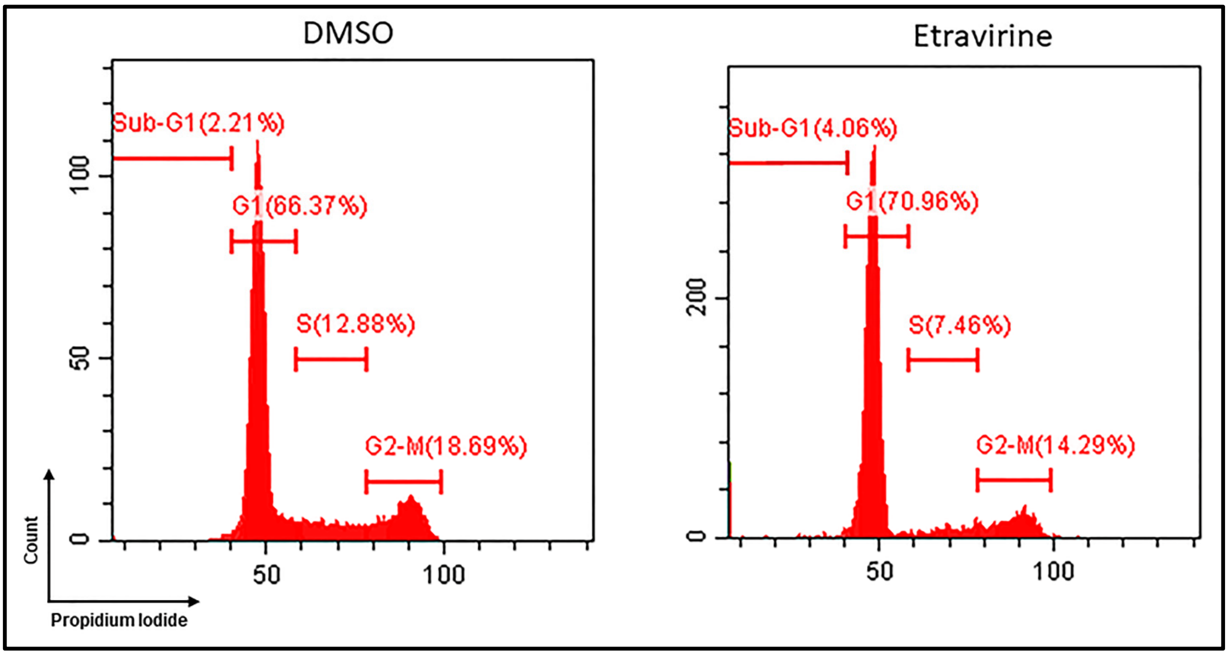

Figure S2: Cell cycle analysis of T47D cells after treatment with etravirine (10 µM) for 48h. Representative histograms with DNA content are shown.

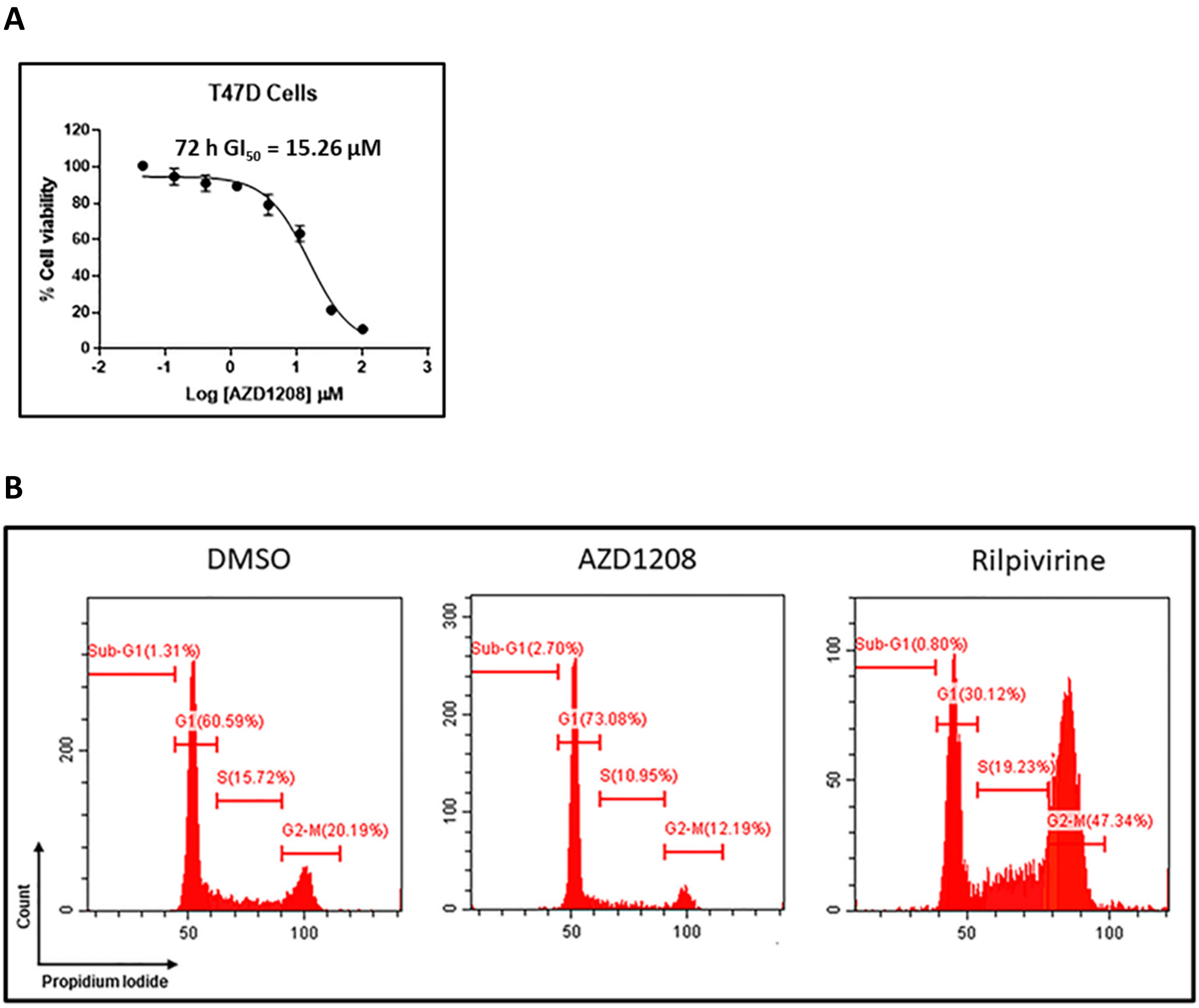

Figure S3: (A) Dose-response curve for AZD1208 against T47D cells at 72 hours. GI_{50} value (µM) is shown. (B) Cell cycle analysis of T47D cells after incubation with AZD1208 (30 µM) or rilpivirine (10 µM) for 48h. Representative histograms with DNA content are shown.

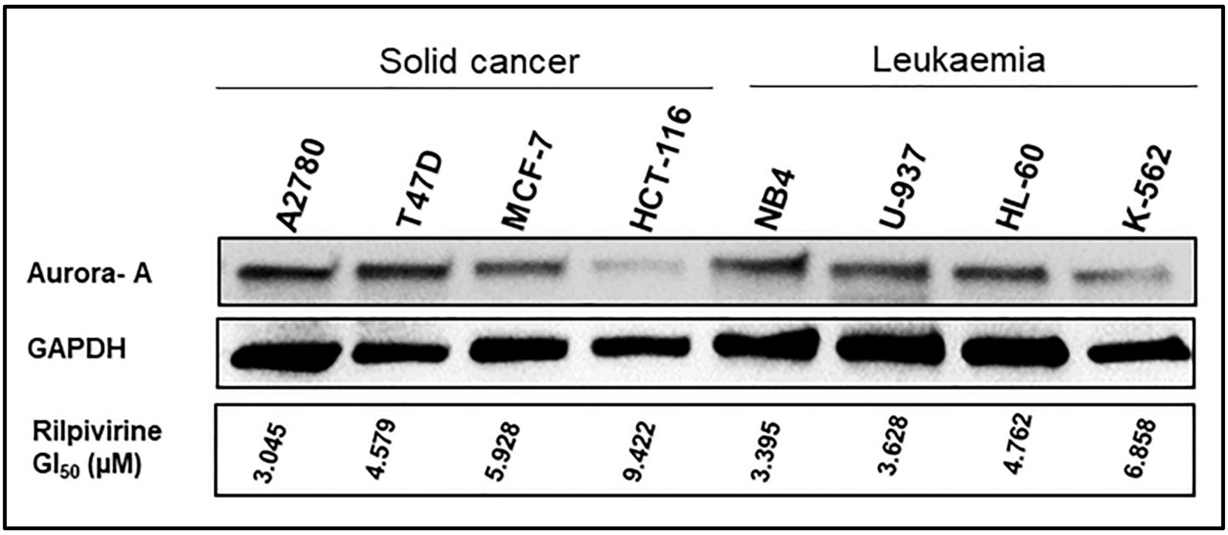

Figure S4: Aurora A protein expression across a panel of solid cancer and leukaemia cell lines with varying sensitivity to rilpivirine, as shown by western blot analysis of cell lysates and GI_{50} values from 72-hour MTT and resazurin assays, respectively.
